# Retroperitoneal fibrous tumor recurring as lung metastases after 10 years: a case report

**DOI:** 10.1186/s40792-021-01209-4

**Published:** 2021-05-22

**Authors:** Kozue Matsuishi, Kojiro Eto, Atsushi Morito, Hirokazu Hamasaki, Keisuke Morita, Satoshi Ikeshima, Kei Horino, Shinya Shimada, Hideo Baba

**Affiliations:** 1 Department of Surgery, Japan Community Health Care Organization Kumamoto General Hospital, 10-10 Toricho, Yatsushiro, Kumamoto 866-8660 Japan; 2 Department of Gastroenterological Surgery, Graduate School of Medical Sciences, Kumamoto University, 1-1-1 Honjo, Chuo-ku, Kumamoto, 860-8556 Japan

**Keywords:** Malignant solitary fibrous tumor, Retroperitoneum, Histopathology

## Abstract

**Background:**

Solitary fibrous tumor (SFT) is a relatively rare mesenchymal tumor that mainly affects adults. Its prognosis is good after curative resection, but distant recurrences after 10 years or longer have been reported. Recurrent SFT usually arises as a local lesion; distant metastasis is rarely reported. Here, we report lung metastases that recurred a decade after excising a retroperitoneal primary SFT.

**Case presentation:**

A 44-year-old woman had an SFT resected from her right retroperitoneum at our hospital. Ten years later, at age 54, she underwent a lung resection after CT showed three suspected metastases in her left lung. All three were histologically diagnosed as lung metastases from the retroperitoneal SFT. However, whereas the primary SFT had 1–2 mitotic cells/10 high power fields (HPF), the metastatic lesion increased malignancy, at 50/10 HPF.

**Conclusion:**

Patients who have had resected SFTs should be carefully followed up, as malignancy may change in distant metastasis, as in this case.

## Background

Solitary fibrous tumor (SFT) is a spindle-cell neoplasm with a varied presentation. It is a rare disease, occurring in only 2.8/100,000 people. SFTs usually develop in the pleura, but 30–40% of SFTs arise in extra-pleural regions [1]. Few reports on retroperitoneal SFT are available. Its prognosis is good after curative resection. However, in rare instances, even benign SFTs can recur as distant metastases long after the primary is resected—sometimes after 10 years or more [2]. Here, we report a case of lung metastasis found 10 years after excision of the primary retroperitoneal SFT.

## Case presentation

**Fig. 1:**
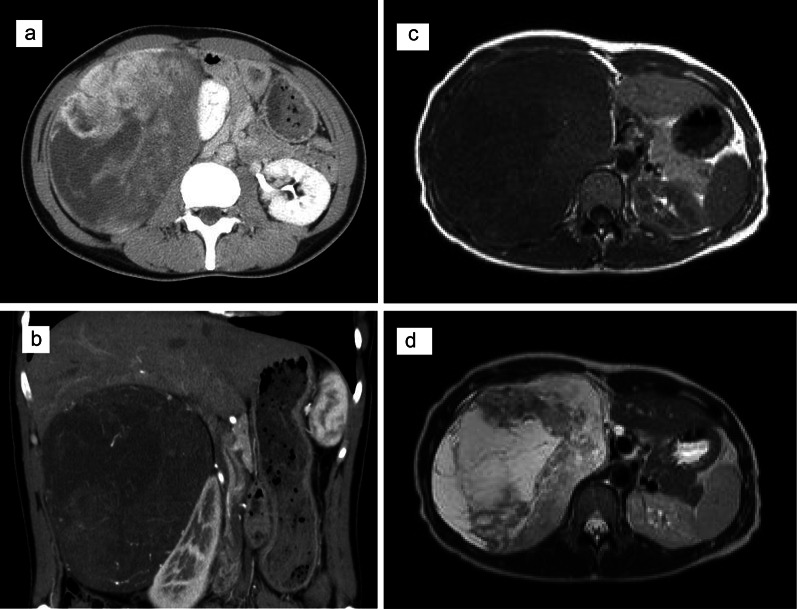
CT and MRI imaging before treatment of primary tumor. **a** Abdominal CT shows a solid encapsulated mass with nonuniform internal structure approximately 16 × 16 × 10 cm, compressing the right kidney and duodenum to the caudal side; **b** contrast-enhanced CT coronary cut; **c** MRI shows association-clear, T1-weighted tumors with predominantly low to equal signals; **d** T2-weighted image showed a high signal intensity tumor with low signal intensity area inside

A 44-year-old woman presented with a chief complaint of abdominal distension. Her primary physician found a retroperitoneal tumor on palpation. She had no medical history and no comorbidities. Laboratory tests showed no significant abnormalities in total blood count, inflammation, liver function, renal function, electrolytes, or coagulation. Various hormone tests were all negative. Computed tomography (CT) showed a 16 × 16 × 10-cm tumor in the right retroperitoneum, which ventrally displaced the duodenum and right kidney (Fig. 1a, b). Magnetic resonance imaging (MRI) also showed the tumor in the retroperitoneum, with a low signal at T1 (Fig. 1c), and a partial high signal at T2 (Fig. 1d).

**Fig. 2:**
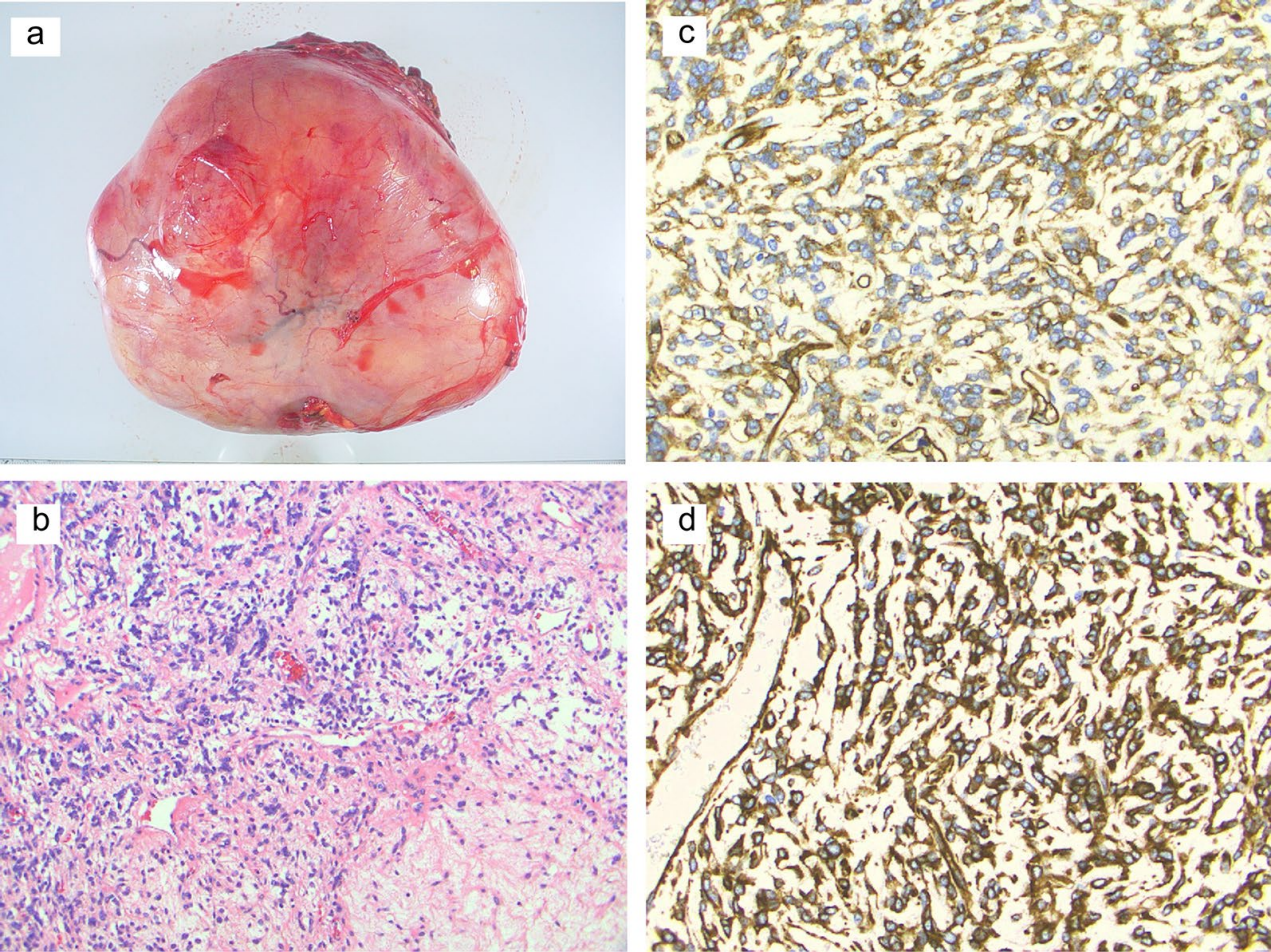
Surgical specimen and histological findings (HE and immunohistochemical staining). **a** Macroscopic findings showed a well-circumscribed and encapsulated elastic hard tumor, 16 × 16 × 10 cm; **b** tumor consisted of spindle-shaped cells with a patternless arrangement and hemangiopericytomatous appearance (×200); **c**, **d** immunohistochemical stains were positive for **c** CD34, **d** and vimentin

No distant metastases were found, and the retroperitoneal mass was curatively resected by laparotomy. The tumor was detachable from the right adrenal gland and other organs, and was judged to be a retroperitoneal tumor. The specimen was 20 cm in size, elastic with soft tissue (Fig. 2a), and had a solid part and a cystic part. Histologically, the tumor consisted of spindle-shaped cells with a patternless pattern and hemangiopericytomatous appearance (Fig. 2b). The cells immunostained positive for CD34 (Fig. 2c) and vimentin (Fig. 2d) and negative for c-KIT, EMA, Desmin, α-SMA and S-100. No necrotic tissue was seen; the specimen showed 1–2 mitotic cells/10 high power fields (HPF). No tumor cells were found on the surgical margin. The tumor was diagnosed as a morphologically and immunologically benign SFT of the retroperitoneum.

**Fig. 3:**
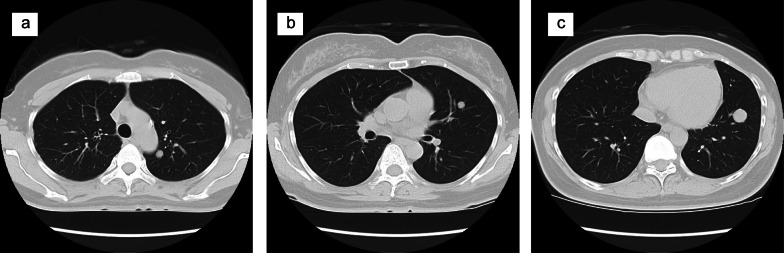
CT imaging of metastases, 10 years after first resection. **a** CT shows 8-mm nodular shadow on the upper left lobe; **b** another 8-mm nodular shadow on the upper left lobe; **c** 15-mm nodular shadow on the lower left lobe

**Fig. 4:**
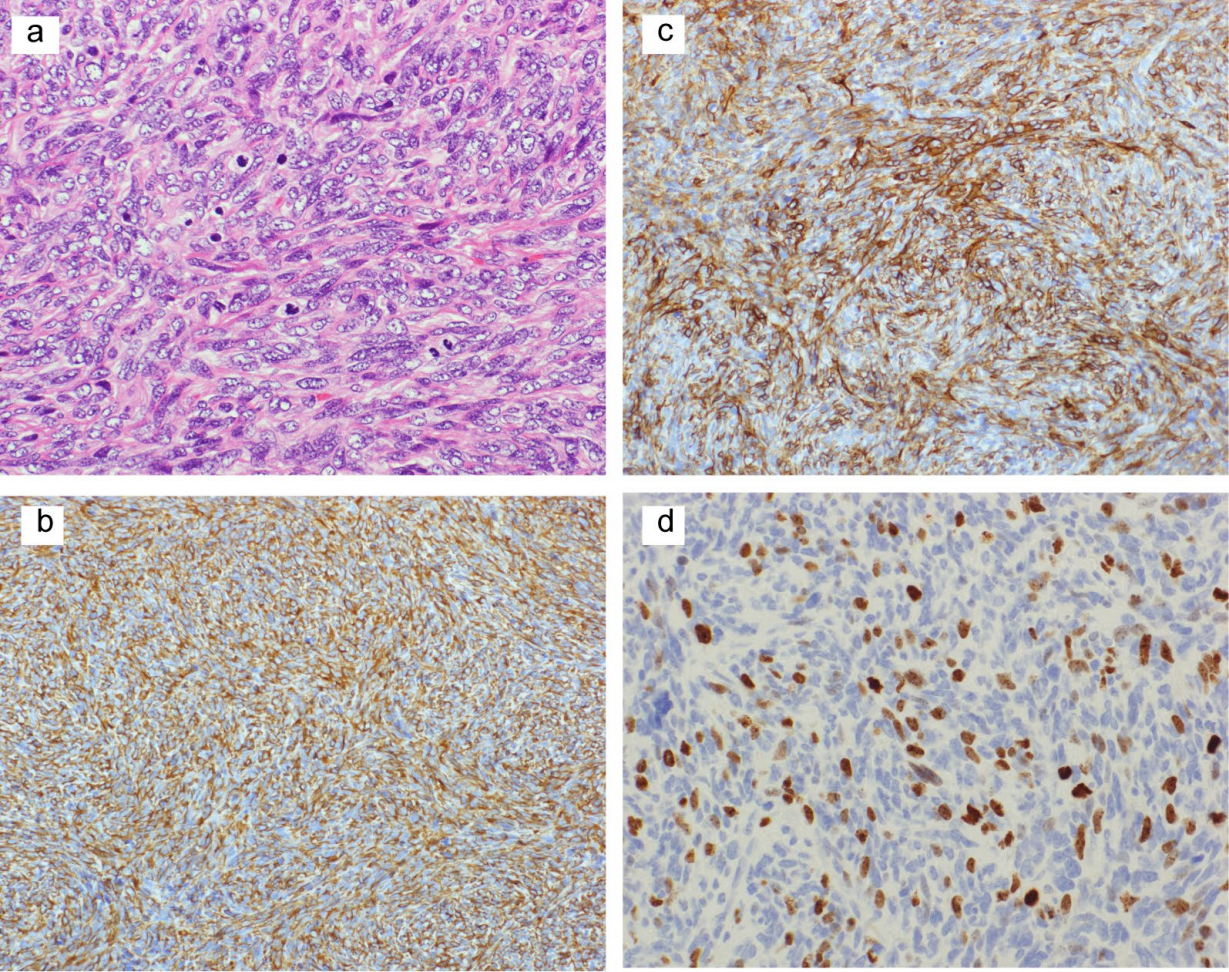
Histological findings (HE and immunohistochemical staining). **a** Lung tumors consisted of spindle-shaped cells with a patternless arrangement and hemangiopericytomatous appearance (×200); **b**, **c** immunohistochemical stains were positive for **b** CD34 and **c** vimentin; **d** MIB-1 index was 20%

The tumor was diagnosed as benign, but because of its huge size, CT scans were performed every 6 months for 5 years. Ten years after resection, a CT scan incidentally revealed metastasis lesions. A CT scan found three nodular shadows in her left lung, including a 15-mm nodular shadow on the lower left lobe, and contrast effects indicating 9-mm and 8-mm margins on the upper lobe (Fig. 3a–c). They were suspected to be metastases of the SFT. She underwent a thoracoscopic-assisted partial left lower lobectomy and an upper left lobectomy. As with the SFT, the resected lung nodules exhibited a patternless pattern (Fig. 4a), positive immunostaining for CD34 (Fig. 4b) and vimentin (Fig. 4c), and negative stains for other interstitial markers. No evidence was seen to contradict these tumors as metastases of the retroperitoneal SFT. However, for the metastatic specimen, the mitotic figure count was 50/10 HPF, and the MIB-1 index was 20% (Fig. 4d), indicating much greater malignant potential. Six months have passed, since the lung metastasis was resected, but she is alive without recurrence.

## Discussion

SFT is an uncommon soft-tissue neoplasm that was first reported as a type of pleural neoplasm in 1931 [3]. SFT derives from fibroblastic or myofibroblastic cells under the mesothelium [4]. It occurs mainly in the thoracic cavity, and rarely, in the retroperitoneal region, as in this case. Simple excision is considered curative for benign SFTs, and is recommended to prevent malignant transformation and metastasis [5]. About 15% of resected SFTs recur, mostly as local metastases. Distant recurrences are rare, but may appear a long time after the primary tumor has been removed [6].

Microscopically, SFTs show multiplying spindle cells with a patternless arrangement, and hemangiopericytoma-like appearance with prominent vascularity [7]. Among immunostains, CD34 is especially useful for differentiating SFT from the other spindle-cell neoplasia [5, 6]. England et al. described high cellularity, high mitotic activity (more than 4/10 HPF), pleomorphism, necrosis, and hemorrhagic changes as criteria for morphological malignancy of SFT [5].

Although SFTs are typically benign, they can become malignant, especially if they grow to a large size or repeatedly recur [1, 8, 9]. According to England’s criteria, the current case was also diagnosed as benign at the initial resection, but the metastases were malignant. This change in malignancy bears concern. Although the primary tumor was considered benign, it potentially harbored highly malignant cells. SFT is often a large tumor, and pathological evaluation of all its cells is difficult; therefore, in an otherwise benign SFT, the possibility of malignant cells, or benign cells that could change morphologically over time, cannot be easily ruled out. Because the tumor was large in this case, we followed up with CT every 6 months for 5 years according to the malignant tumor. Lung metastasis has been pointed out by CT 10 years later coincidentally. Currently, there are no rules regarding follow-up. However, we think that it may be better to follow up for 10 years, because there is a possibility of distant recurrence like this time. However, accumulation of more cases is necessary to better understand this rare tumor.

## Conclusion

Patients who have had resected SFTs should be carefully followed up, as malignancy may change in distant metastasis, as in this case.

## Data Availability

All data generated or analyzed during this study are included in this published article.

## Abbreviations

CT: Computed tomography
MRI: Magnetic resonance imaging
SFT: Solitary fibrous tumor
